# Epidermal growth factor prevents *APOE4* and amyloid-beta-induced cognitive and cerebrovascular deficits in female mice

**DOI:** 10.1186/s40478-016-0387-3

**Published:** 2016-10-27

**Authors:** Riya Thomas, Paulina Zuchowska, Alan W. J. Morris, Felecia M. Marottoli, Sangeeta Sunny, Ryan Deaton, Peter H. Gann, Leon M. Tai

**Affiliations:** 1 Department of Anatomy and Cell Biology, University of Illinois at Chicago, Chicago, IL 60612 USA; 2 Research Histology and Tissue Imaging Core (RHTIC), University of Illinois at Chicago, Chicago, IL 60612 USA; 3 Department of Pathology, University of Illinois at Chicago, Chicago, IL 60612 USA

**Keywords:** Epidermal growth factor, Alzheimer’s disease, Apolipoprotein E4, Cerebrovasculature

## Abstract

Cerebrovascular (CV) dysfunction is emerging as a critical component of Alzheimer’s disease (AD), including altered CV coverage. Angiogenic growth factors (AGFs) are key for controlling CV coverage, especially during disease pathology. Therefore, evaluating the effects of AGFs in vivo can provide important information on the role of CV coverage in AD. We recently demonstrated that epidermal growth factor (EGF) prevents amyloid-beta (Aβ)-induced damage to brain endothelial cells in vitro. Here, our goal was to assess the protective effects of EGF on cognition, CV coverage and Aβ levels using an AD-Tg model that incorporates CV relevant AD risk factors. *APOE4* is the greatest genetic risk factor for sporadic AD especially in women and is associated with CV dysfunction. EFAD mice express human *APOE3* (E3FAD) or *APOE4* (E4FAD), overproduce human Aβ42 and are a well characterized model of *APOE* pathology. Thus, initially the role of *APOE* and sex in cognitive and CV dysfunction was assessed in EFAD mice in order to identify a group for EGF treatment. At 8 months E4FAD female mice were cognitively impaired, had low CV coverage, high microbleeds and low plasma EGF levels. Therefore, E4FAD female mice were selected for an EGF prevention paradigm (300 μg/kg/wk, 6 to 8.5 months). EGF prevented cognitive decline and was associated with lower microbleeds and higher CV coverage, but not changes in Aβ levels. Collectively, these data suggest that EGF can prevent Aβ-induced damage to the CV. Developing therapeutic strategies based on AGFs may be particularly efficacious for *APOE4*-induced AD risk.

**Electronic supplementary material:**

The online version of this article (doi:10.1186/s40478-016-0387-3) contains supplementary material, which is available to authorized users.

## Introduction

Cerebrovascular (CV) dysfunction is re-emerging as a critical component of Alzheimer’s disease (AD) (reviewed in [36]). An unresolved issue is whether AD pathways induce detrimental effects on CV length and the significance for cognitive dysfunction. There is evidence of both higher [7, 9, 13] and lower [18, 25, 29, 39] vessel length and/or density (vessel coverage) in AD-patients and mice (AD-Tg) that overproduce an important contributor to AD progression; human amyloid-beta (Aβ). Angiogenic growth factors (AGFs) are key for controlling total vessel coverage. Therefore one approach for addressing issues in the field is evaluating the effects of AGFs on AD-like pathology in vitro and in vivo. Positive in vivo effects would also support future therapeutic research focused on developing AGF-like drugs for AD. Despite the promise of AGFs in models of stroke, published data in AD models are limited and for some AGFs equally as conflicting as the data on CV coverage in AD [14, 40, 44, 47], with the possible exception of the vascular endothelial growth factor (VEGF). Peripheral VEGF administration [58] or neuronal overexpression [44] improves cognition and lowers Aβ levels in AD-Tg mice. Beyond these reports review or opinion articles form the basis of our knowledge of AGFs in AD [3, 26, 54]. We recently compared the activity of the main AGFs at preventing Aβ-induced vessel degeneration in vitro [31]. Epidermal growth factor (EGF) prevented oligomeric Aβ42 (oAβ)-induced angiogenesis deficits and disruption of preformed tubes. EGF was also more protective than other AGFs, including VEGF. The role of EGF in AD is unclear as both higher and lower plasma EGF levels have been reported in AD patients [8, 17, 24, 30]. More recently low EGF levels were reported to predict the conversion from amnestic mild cognitive impairment to AD [33], supporting the hypothesis that exogenous EGF will be protective in AD-Tg mice. Contrastingly, there is improved cognition in AD-Tg mice treated with the EGF receptor inhibitor gefitinib [57]. Therefore, the primary goal of this study was to assess the protective effects of EGF on cognition, CV coverage and Aβ-levels using an AD-Tg model that incorporates CV relevant AD risk factors.

*APOE4* is the greatest genetic risk factor for sporadic AD, increasing risk up to 12-fold compared to *APOE3* (reviewed in [34]). *APOE4*-induced AD risk is also greater in females [2, 10, 41]. As *APOE4* carriers also often respond differentially in clinical trials it is important to identify novel mechanistic processes underlying *APOE4*-induced AD risk. Increasing evidence indicates that *APOE* modulates CV morphology and function (reviewed in [50]). In AD and AD-Tg models *APOE4* is associated with CV dysfunction including basement membrane degradation and increased leakiness [50]. Thus, incorporating human *APOE4* in studies focused on the CV in AD is valuable.

EFAD mice express human *APOE3* (E3FAD) or *APOE4* (E4FAD), overproduce human Aβ42 and are a well characterized model of *APOE* modulated Aβ42 pathology [61]. We initially assessed the role of *APOE* and sex in cognitive and CV dysfunction in EFAD mice in order to identify a group for EGF treatment. E4FAD female mice were cognitively impaired, had high microbleeds, low CV coverage and low plasma EGF levels at 8 months of age. Therefore, E4FAD female mice were selected for an EGF prevention paradigm (300 μg/kg/wk, 6 to 8.5 months). EGF prevented cognitive decline and was associated with lower microbleeds and higher CV coverage, but not with changes in Aβ levels. Collectively these data suggest that EGF can prevent Aβ-induced damage to the CV. Developing therapeutic strategies based on AGFs may be particularly efficacious for *APOE4*-induced AD risk.

## Materials and methods

### Experimental design

All protocols follow the UIC Institutional Animal Care and Use Committee protocols. Breeding and colony maintenance was conducted at the UIC as described in [61]. EFAD mice express five Familial Alzheimer’s disease (FAD) mutations (APP K670N/M671L + I716V + V717I and PS1 M146L + L286V) and human *APOE3* (E3FAD mice) or *APOE4* (E4FAD mice) i.e. 5xFAD^+/−^ *APOE* ^+/+^. EFAD mice utilized to start our breeding colonies were kindly provided by Dr. M.J.Ladu.

#### Evaluation of cognitive and CV changes in EFAD mice at 8 months

Male (EFADM) and female (EFADF) E3FAD and E4FAD mice at 8 months of age were sequentially assessed (24 hrs. between tests) using the open field, novel object recognition and spontaneous alternation (Y-maze) tests. EFAD mice were then injected with sodium fluorescein (NaFl, Sigma), anesthetized (100 mg/kg ketamine and 10 mg/kg xylazine), blood drawn via cardiac prick and transcardially perfused with PBS containing protease inhibitors (Millipore). Right hemi-brains were dissected into the cortex, hippocampus, and cerebellum and processed for NaFl extravasation. Left hemi-brains were frozen over dry ice in cryomolds containing O.C.T compound (Tissue-Tek), and stored at −80 °C until processing for immunohistochemical (IHC) analysis. All investigators were blinded for *APOE* genotype. *n* = 7 (E3FADM), 7 (E3FADF), 9 (E4FADM), 8 (E4FADF).

#### Treatment of female E4FAD mice with EGF

Six month old E4FADF mice were administered EGF (Shenandoah, 300 μg/kg/wk) or vehicle control (water) by intraperitoneal injection (i.p.) until 8.5 months. Body weights were monitored prior to every injection. Mice were sequentially assessed (24 hrs. between tests) using the open field, novel object recognition, spontaneous alternation (Y-maze) and novel arm entry (Y-maze) tests. At the end-point behavior was further assessed using the light-dark box and Morris water maze tests and food consumption monitored over 24 h. 30 min prior to sacrifice mice were injected with EGF, dissected right hemi-brains stored at −80 °C until homogenization and left hemi-brains were stored for h﻿istological analysis. NaFl extravasation was not conducted on the EGF or VC treated E4FADF mice to enable biochemical analysis of Aβ, apoE and EGF levels in brain homogenates. For all analysis investigators were blinded for treatment. *n* = 8 per group.

### Behavioral analysis

Behavioral analysis was conducted in the mouse dark cycle, tracked in real time by an overhead camera and videos analyzed using the ANY-Maze software.

Open field. Mice were placed in the center of a white box (l38.5xw30xh30 cm) and allowed to freely move for 10 min. The distance traveled and average speed were measured.

**Fig. 1:**
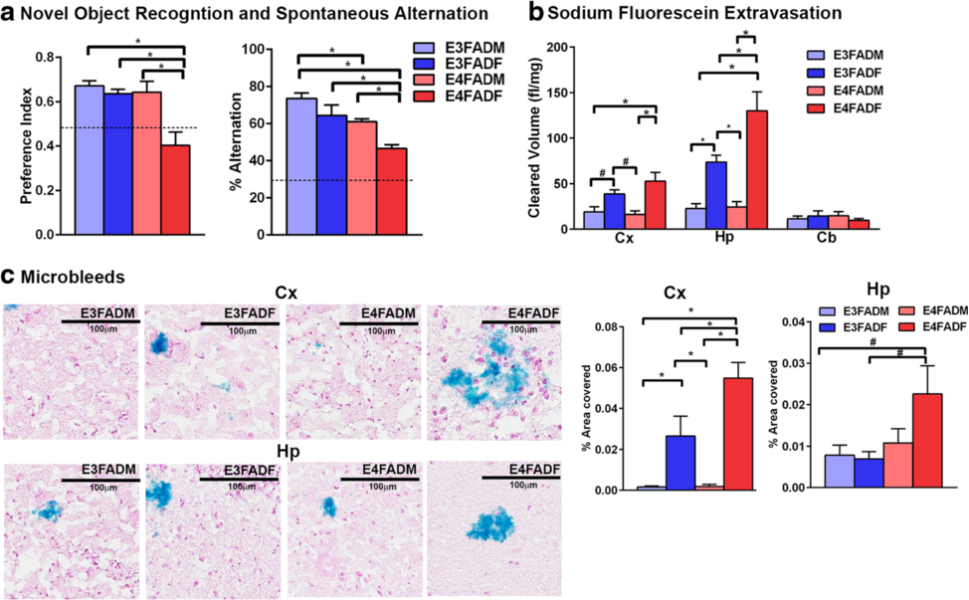
Cognitive and cerebrovascular dysfunction in E4FADF mice. **a** E4FADF mice are cognitive impaired when assessed by novel object recognition and spontaneous alternation. **b** There are higher NaFl levels in the cortex (Cx) and hippocampus (Hp) of E4FADF mice after i.p injection. *n* = 7(E3FADM), 6 (E3FADF), 9 (E4FADM), 7 (E4FADF). **c** Prussian blue staining (microbleeds) is higher in the Cx and Hp of E4FADF mice when assessed by IHC analysis. *n* = 7 (E3FADM), 7 (E3FADF), 9 (E4FADM), 8 (E4FADF). Data expressed as mean +/− S.E.M. *p < 0.05 by two-way ANOVA and Tukey’s post hoc comparisons. #*p* < 0.05 by two-way AVOVA followed Fisher’s LSD test

Novel object recognition was conducted as described in [62] with slight modifications. On day 1, mice were habituated in a white testing chamber (38.5x30x30 cm) for 20 min. On day 2, mice were placed in the same testing chamber that contained two identical objects for 7 min, returned to their home cage for 1 hr and placed in the testing chamber with a familiar and a novel object for 7 min. Time spent with each object and the preference index (ratio of time spent with the novel object divided by total investigation time for both objects) were calculated. Mice that did not investigate both objects for longer than 5 s were not included. Data for one E3FADF and one E4FADF were removed (Fig. 1) based on the exclusion criteria.

#### Spontaneous alternation (Y-maze)

Mice were placed in one arm of a Y-maze apparatus (38.5x8x13 cm, spaced 120° apart) allowed to explore for 7 min and the sequence of arm entries were recorded. Spontaneous alternation was calculated as the number of alternations (entries into three different arms consecutively) divided by the total possible alternations (the number of arms entered minus 2) and multiplied by 100.

#### Novel arm entry (Y-maze)

Mice were placed into the maze (plus extra-maze cues) with one of the arms blocked for 10 min, returned to the home cage for 60 min and placed back in the maze with access to all three arms for 5 min. The time spent in the novel arm was calculated.

#### Light-dark box

The light-dark box (21x42x25 cm) consists of two chambers with an opening in-between (7.3 cm): the light chamber is brightly illuminated with white walls (66.6 % of the box) the dark chamber comprises of dark walls (33 %). Mice were placed into the light side and allowed to move freely for 5 min. The time spent in each chamber was recorded.

Morris water maze (MWM) was conducted as described in [35] with slight modifications in three phases. The circular pool was 120 cm in diameter and 50 cm tall, and the circular escape platform was 10 cm in diameter. The pool was filled with water containing non-toxic tempera paint (maintained at 25 °C) to 10 cm below the top rim and divided into equal-sized imaginary quadrants. Extramaze cues were placed in the four corners for spatial orientation. A single mouse was in the pool for each testing phase/session. MWM testing was comprised of three phases. (a) Visual Cue phase. Mice were trained over the course of 2 days to locate a flagged hidden platform (60s trial time, four trials each day with a 20 min inter-trial interval (ITI)). (b) Acquisition phase. After 2 days, mice were trained for 5 days (60s trial time, four trials each day with a 20 min ITI) to locate the position of the hidden platform (remains on the hidden platform for >2 s). During the visual cue and acquisition phases the mouse’s entry quadrant varied but the platform location remained constant. Latency to find the platform (s) was measured. (c) Probe trial. 1 h following the final acquisition trial, a single 60s probe trial was conducted with the platform removed. The latency to the target area (i.e., where platform was located during acquisition phase) and the time spent in the target quadrant were calculated.

### Sodium fluorescein extravasation

Mice were injected i.p. (200 μl) with 2 % NaFl, sacrificed after 30 min and tissue harvest conducted as described above. Dissected brain regions were weighed, homogenized in PBS, suspended in equal volumes of 60 % tricholoroacetic acid, then vortexed and centrifuged (18,000 × *g*,10 min, 4 °C). Fluorescence levels were measured using a microplate reader (SpectraMax i3x, Molecular Devices) and cleared volumes calculated as: [1/plasma levels (fluorescence units/μl) x total brain fluorescence]/brain weight.

### Biochemical analysis

Dissected brains were weighed and sequentially extracted using the 3-step extraction protocol as described in [61].

#### Protein quantification

Total protein in Tris-buffered saline (TBS) and neutralized formic acid (FA) extracts was quantified using the Ready to Use Bradford Reagent (Bio-Rad) and for TBS-Triton X100 (TBSX) extracts the BCA Protein Assay Kit (Pierce).

For western blot analysis of TBS-X fractions protein (20 μg) was separated on 4–12 % Bis-Tris gels (Invitrogen) and transferred onto low-fluorescence PVDF membranes. Membranes were blocked (1 hr. room temperature) with 5 % non-fat milk in TBS containing 0.1 % Tween 20 (TBST), then incubated (4 °C, overnight) with primary antibodies against post synaptic density protein (PSD-95, 1:1000, Cell Signaling), synaptophysin (1:1000, Cell Signaling), the EGF receptor (1:100, Santa Cruz Biotechnology) or actin (1 hr. room temperature, 1: 20,000, Cell Signaling) in TBST with 5 % bovine serum albumin (BSA). Membranes were incubated in secondary antibodies (Jackson Immunoresearch) with TBST wash (3X5min) in between steps and imaged using an Odyssey ® Fc Imaging System. Image J was utilized to quantify actin normalized proteins.

#### ELISA

ApoE and Aβ42 (Life Technologies) levels were measured in the TBS, TBSX and FA extracts, and oAβ (Biosensis) in the TBS extracts. The apoE ELISA utilizes α-apoE (1:2000, Millipore) capture and α-apoE biotin-gt (1:5000, Meridian) detection antibodies as described in [48]. EGF levels were measured in the plasma and TBS extracts (R&D).

### Histological analysis

Frozen brains were sectioned at 12 μm and fixed using 10 % Neutral Buffered Formalin (Sigma). Nine nonadjacent sections (108 μm apart) were utilized for quantification per animal.

#### Prussian blue stain

Slides were hydrated in distilled water, submerged in equal parts of potassium ferrocyanide and hydrochloric acid (30 min), rinsed in distilled water (1 min) and counter-stained with Nuclear Fast Red (5 min, Rowley Biochemical Institute J-606-3). After another wash in distilled water (1 min) slides were dehydrated (50, 75, 95, & 100 % alcohol series), cleared in xylene and mounted. Slides were scanned at 20x magnification on a Leica Aperio AT2 scanner. The resulting image files were loaded into Aperio eSlide Manager (version 12.3.1.6002) and regions of interest were drawn using Aperio ImageScope (version 12.3.2.5030) around the cortex and hippocampus in separate layers and scored using Definiens Tissue Studio (version 4.2) using the ‘Marker Area’ solution. The solution was modified to identify Nuclear Fast Red and quantify the percent of tissue positive for Prussian blue (indicating microbleeds) in each of the brain regions. Data for multiple sections per brain were combined.

#### CD31 immunostain

Sections were incubated in 0.3 % hydrogen peroxide (30 min), blocked in 10 % rat-serum (2 hr) and incubated with a rat anti-mouse CD31 antibody (MEC13.3, BD Bioscience 1:10, 4 °C overnight). Sections were stained with the Vectastain ABC Kit according to the manufacturer’s protocol (Vector), dehydrated through alcohol series and cleared in xylene with PBS (3X5min) washes in between steps. Slides were digitally scanned and traced (deep layer cortex and subiculum) as for Prussian blue. The Aperio microvessel algorithm (version 11.2.0.780) was optimized to quantify the percent area of tissue and density occupied by blood vessels per brain region on the slide.

#### Laminin and Aβ stain

Slides were incubated in 60 % FA (8 min), sections permeabilized with TBS containing 0.25 % TBSX (3X5min) and blocked with 5 % BSA in TBSX (2 hr). Slides were incubated with anti-Aβ (MOAB-2, mouse IgG_2b_, 1:400 dilution, Biosensis) and anti-laminin (Rabbit, 1:400 dilution, Abcam) antibodies in TBS containing 2 % BSA and 0.10 % Triton X-100 (4 °C, overnight) in a humidified chamber. Slides were washed (3x5min in TBSX), incubated with Alexa fluorophore-conjugated secondary antibodies (AlexaFlour 750 anti-rabbit, Alexafluor 350 anti-mouse IgG_2b_) diluted 1:200 in TBSX containing 2 % BSA (2 hr) and washed in TBSX (3X5min) followed by TBS (1x5min). Mosaic and single images were captured on a Zeiss Axio Imager M1 under identical capture settings at 20x magnification. Converted images were thresholded equally to diminish background signal (NIH ImageJ software). Identified objects after thresholding were scanned to validate either vessel or Aβ staining. Mosaic images were outlined for the subiculum, deep layer or full cortex and quantified (Analyze Particles function).

#### Thioflavin-S stain

Slides were incubated in filtered 1 % aqueous Thioflavin-S (5 min, Sigma) protected from light, dehydrated in 70 % ethanol (2X5min) and washed in PBS (2X2min). Mosaic images were captured as for laminin and MOAB-2 staining at 10X magnification. Plaques were evaluated for total number and percent area covered (total Thioflavin-S immunoreactivity/ROI) using Image J as described in [61].

#### Claudin 5 stain

A representative slide from EGF or VC treated E4FADF mice was stained according to the protocol described for Laminin staining, using an anti-claudin 5 antibody (1:20, Santa Cruz Biotechnology) and AlexaFlour 647 anti-rabbit secondary antibody (1:200).

### Statistical analysis

All data are presented as mean +/−S.E.M, and were analyzed using two-way ANOVA followed by either Tukey’s, Fisher’s LSD or Sidak’s post hoc comparisons or using Student’s *t*-test, as specified in the figure legends using GraphPad Prism version 6.

## Results

### Female E4FAD mice are cognitively impaired at 8 months of age

#### EFAD mice overproduce Aβ42 (via 5XFAD mutations)

APP K670N/M671L + I716V + V717I and PS1 M146L + L286V) and express human *APOE3* (E3FAD mice) or *APOE4* (E4FAD mice). In EFAD mice Aβ pathology is prevalent in the hippocampal formation, primarily the subiculum, and in the deep layers of the frontal cortex [61]. Published data demonstrate that by 7 months soluble Aβ and extracellular plaque levels follow the order: E4FAD female (E4FADF) > E3FAD female (E3FADF) = E4FAD male (E4FADM) > E3FAD male (E4FADM) [12]. Specific for the CV, cortical microbleeds are also reported as highest in E4FADF mice [12]. In order to select a sex and genotype of EFAD mice for treatment with EGF, it was important to identify the association among cognitive decline and CV dysfunction. Initially, cognitive impairment was assessed in E3FADM, E3FADF, E4FADM and E4FADF mice at 8 months of age. E4FADF mice were cognitively impaired compared to all other groups when assessed by both novel object recognition and spontaneous alternation (Y-maze) tests (Fig. 1a, two-way AVOVA followed by Tukey’s post-hoc analysis). Indeed, E4FADF mice performance was ~35 % lower in novel object recognition and ~30 % lower in the spontaneous alternation test.

### Higher CV leakiness and lower CV coverage in E4FADF mice

To determine the role of *APOE* and sex in CV leakiness, brain levels of NaFl were assessed after i.p. injection (Fig. 1b). NaFl levels were significantly higher in the cortex of E4FADF mice compared to E3FADM and E4FADM mice and were higher in hippocampus compared to all other groups (two-way AVOVA followed by Tukey’s post-hoc analysis). NaFl levels were also higher in E3FADF mice compared to EFADM mice (two-way AVOVA followed by Tukey’s post-hoc analysis) in the hippocampus, whereas in the cortex these effects were only significant by two-way AVOVA followed by Fisher’s LSD test. Prussian blue staining was conducted to assess microbleed coverage as a complimentary measure of CV leakiness (Fig. 1c). In the cortex the area of microbleed coverage followed the order E4FADF > E3FADF > E4FADM = E3FADM (Fig. 1c, two-way AVOVA followed by Tukey’s post-hoc analysis). In general cortical microbleed coverage was 20-fold higher in E3FADF mice and 55-fold higher in E4FADF mice compared to EFADM mice. In the hippocampus microbleed coverage was only higher in E4FADF mice when assessed by two-way AVOVA followed by Fisher’s LSD test.

**Fig. 2:**
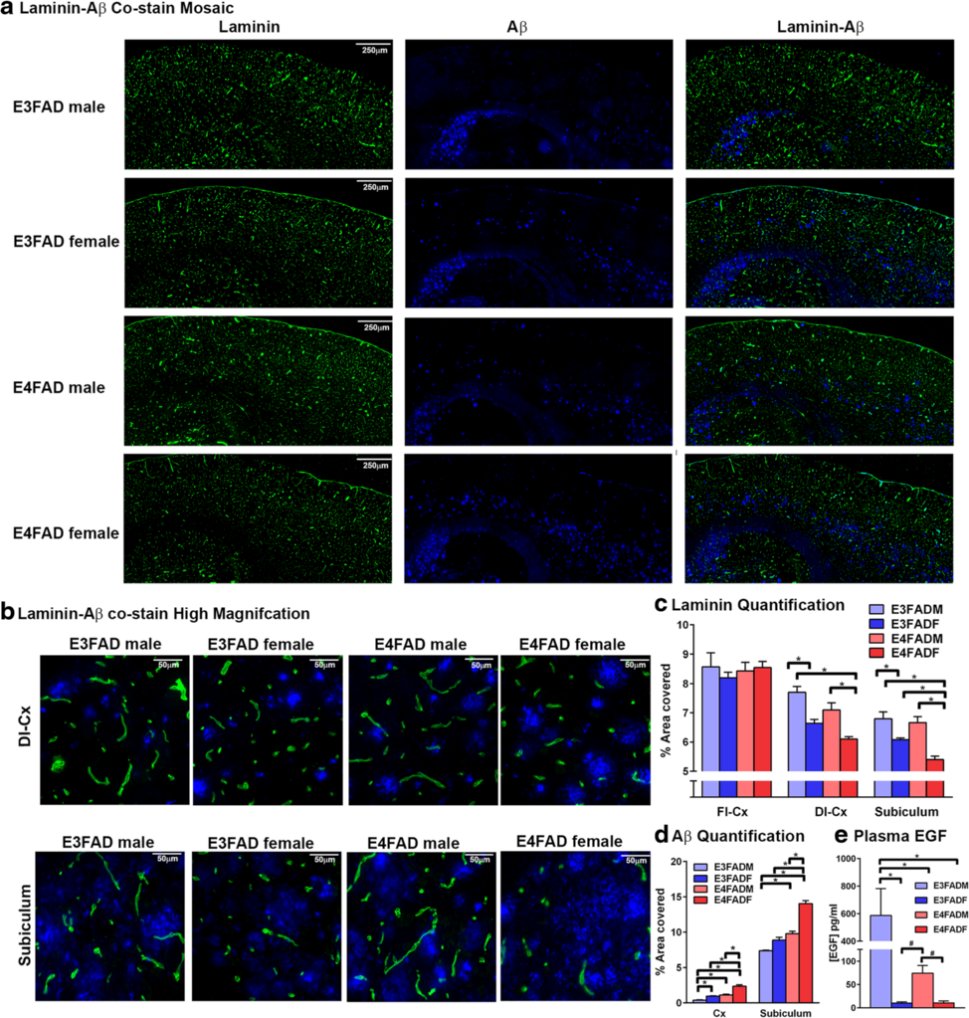
Lower laminin coverage in E4FADF mice. **a**–**c** Laminin (basement membrane protein) staining is lower in E4FADF mice in brain regions of extracellular Aβ pathology: the subiculum and deep layers of the cortex (Dl-Cx) but not significantly different in the full-length cortex (Fl-Cx) between groups when assessed by IHC analysis. Further, E3FADF mice exhibit lower staining than E3FADM mice. **d** Extracellular Aβ levels are highest in E4FADF mice (IHC analysis using the antibody MOAB2). **e** Plasma EGF levels are low in EFADF mice. *n* = 7(E3FADM), 7 (E3FADF), 9 (E4FADM), 8 (E4FADF) when assessed by ELISA. Data expressed as mean +/− S.E.M. *p < 0.05 by two-way ANOVA and Tukey’s post hoc comparisons. ^#^ *p* < 0.05 by Student’s *t*-test

Next, laminin (basement membrane protein)-Aβ co-staining was conducted as an indication of total vessel coverage. Visually laminin staining appeared lower in deeper layers of the frontal cortex and the subiculum in EFADF mice compared to males, an effect particularly pronounced in E4FADF mice (Fig. 2a  and b). When quantified for % area covered (Fig. 2c), no differences were observed for laminin staining among the groups for the whole cortex. In the deep layer cortex laminin staining followed the order: E4FADM/E3FADM > E3FADF > E4FADF (two-way AVOVA followed by Tukey’s post-hoc analysis). Data for the subiculum followed the same pattern. Overall, laminin staining was 10 % lower in E3FADF mice and 20 % lower in E4FADF mice in the deep layer cortex and subiculum compared to EFADM mice. Extracellular Aβ levels followed the order E4FADF > E4FADM = E3FADF > E3FADM (Fig. 2d).

### Plasma EGF levels are lowest in EFADF mice

EGF plays a key role in controlling total vessel coverage and a recent report indicates that low plasma EGF predict the conversion from MCI to AD [33]. We therefore measured plasma EGF levels in EFAD mice (Fig. 2e). E3FADM mice had higher plasma EGF levels than all other groups (2-way ANOVA, followed by Tukey’s post-hoc comparison). There was also a trend of higher EGF plasma levels in E4FADM mice compared to EFADF mice, which was significant by Student’s *t*-test. One caveat is that NaFl injection may have modulated plasma EGF levels in the EFADF mice, in an *APOE* genotype specific manner.

Collectively, these data support that 8 month old EFADF mice are cognitively impaired, have low CV coverage, high CV permeability and low plasma EGF levels. Therefore E4FADF mice were selected for treatment with EGF in a prevention paradigm.

### EGF prevents cognitive decline in E4FADF mice

**Fig. 3:**
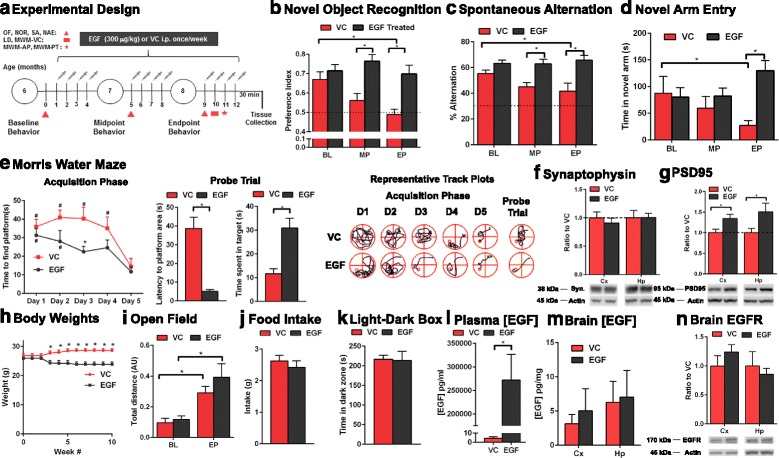
EGF prevents cognitive deficits in E4FADF mice. **a** Experimental design for EGF prevention paradigm in E4FADF mice. EP, end-point; MP, mid-point; EP, end-point; OF, open field; SA, spontaneous alternation; NOR, novel object recognition; NAE, novel arm entry; LD, *light*-*dark* box; MWM-VC, Morris water maze visual cue phase; MWM-AP, Morris water maze acquisition phase; MWM-PT; Morris water maze probe trial. **b**–**d** EGF prevents cognitive decline when assessed by NOR, SA and NAE. *Dashed line* represents no preference (novel object) or chance alternation (spontaneous alternation). **e** EGF treatment improves performance in the Morris water maze test (EP). Representative track plots of probe trial is based on latency to previous platform area. **f** Synaptophysin levels are the same in EGF and VC treated mice when assessed by western blot analysis. **g** PSD-95 levels are higher in EGF treated E4FADF mice (western blot analysis). **h** Body weight decreased at 4 weeks but remained constant until 10 weeks with EGF treatment. **i**–**k** EGF treatment did not modulate performance in open field, had no effects on food intake (over 24 hrs at EP) and did not change performance in the *light*-*dark* box test. **l**, **m** EGF treated mice had higher plasma but not brain levels of EGF (ELISA). **n** There were no changes in EGF receptor levels in the cortex or hippocampus after EGF treatment (western blot). *n* = 8 per group for **a**–**m** and *n* = 6 for **n**. Data expressed as mean +/− S.E.M. *p < 0.05 by 2-way ANOVA and Sidak’s post-hoc analysis (B-E, G and H). *p < 0.05 by Students *t* test (E probe trial, F, I-K)

Our overarching goal was to test EGF in a prevention paradigm based on our recent data that EGF prevents Aβ-induced disruption of angiogenesis and degeneration of preformed tubes in vitro [31]. E4FADF mice were treated with EGF (300 μg/kg/wk) by i.p. from 6 to 8.5 months and tested by a battery of tests for cognitive ability (Fig. 3a). 300 μg/kg/wk was selected as a low dose that does not promote cancer or overt signs of toxicity in mice [6]. EGF induced a profound protective effect on cognitive decline in E4FADF mice compared to the vehicle control (VC). When assessed by novel object recognition (Fig. 3b), spontaneous alternation (Fig. 3c, Y-maze) and novel arm entry (Fig. 3d, Y maze) the VC control groups progressively declined in cognitive ability from 6 to 8 months (two-way ANOVA followed by Sidak’s post hoc comparisons which takes into account same animal re-testing). In contrast, no age-dependent decline was observed for EGF treated mice. At the mid-point EGF treated mice performed 46 % higher in the novel object recognition and 37 % higher in the number of spontaneous alternations performed (Y-maze) compared to the VC. At the end-point, EGF treated mice performed ~58 % higher in novel object recognition and spontaneous alternation and spent 477 % longer in the novel arm test (Y-maze).

Cognitive ability was further dissected at the end-point in the Morris water maze test (Fig. 3e). In the acquisition phase EGF (day 1 vs 5 and 2 vs 5 were significant) and VC treated mice (day 1, 3 and 4 vs day 5 were significant) learned the location of the platform by day 5. On day 3 the EGF treated mice located the platform earlier than the VC, indicative of improved learning. In the probe trial (Student’s *t*-test) EGF treated mice located the previous platform area with a lower latency time (VC = 38.6 s, EGF = 5.15 s) and spent a longer time in the target quadrant (VC = 11.6 s, EGF = 30.9 s). EGF did not modulate levels of the pre-synaptic protein synaptophysin in the cortex or hippocampus (Fig. 3f). However, consistent with the behavior data levels of the postsynaptic protein PSD95 were higher in the cortex (35 %) and hippocampus (50 %) in EGF treated mice (Fig. 3g, Student’s *t*-test). Thus, in a prevention paradigm EGF-induces a beneficial effect on cognition and post-synaptic neuronal markers.

### EGF treatment had no effects on locomotion, food intake, anxiety-like behavior and brain EGF levels

A potential confounding factor for the beneficial effects of EGF on cognition is whether EGF modulates anxiety-like behavior. VC treated mice remained steady in weight until week 4 (7 months old) where they gained ~5 % and then remained at the same weight for the rest of the study. EGF treated mice lost ~ 7 % in weight at week 4 and then remained steady (Fig. 3h). The changes in bodyweight were not related to general locomotion, as in the open field test there were no differences between EGF and VC treated mice (Fig. 3i, two-way ANOVA followed by Sidak’s post hoc). At the end-point (8 months) there were no differences in food intake (Fig. 3j over 24 hrs, Student’s *t*-test) or anxiety like behavior as assessed in the light-dark box test (Fig. 3k, Student’s *t*-test). The improved cognition with EGF compared to the VC was not related to general locomotion or anxiety like effects.

EGF can exert pleiotropic actions on many cells within the brain and it was important to determine whether brain EGF levels were increased after treatment. 30 min prior to sacrifice mice were treated with EGF and levels assessed by ELISA in the plasma (Fig. 3l, Student’s *t*-test), cortex, and hippocampus (Fig. 3m). Plasma EGF levels were ~4 pg/ml in VC treated mice and ~ 250 ng/ml after EGF treatment. There were no differences in hippocampal or cortical EGF levels between VC and EGF treated mice. In addition, when assessed by western blot analysis EGF receptor levels were not altered by EGF treatment in the hippocampus or cortex of E4FADF mice (Fig. 3n and Additional file 1: Figure S1d). However, we cannot exclude that there were cell-specific changes in EGF receptor levels after treatment. Therefore, the beneficial effects of EGF on cognition were likely mediated by peripheral rather than CNS levels of EGF.

### EGF treatment lowers microbleeds and increases CV coverage compared to the VC

**Fig. 4:**
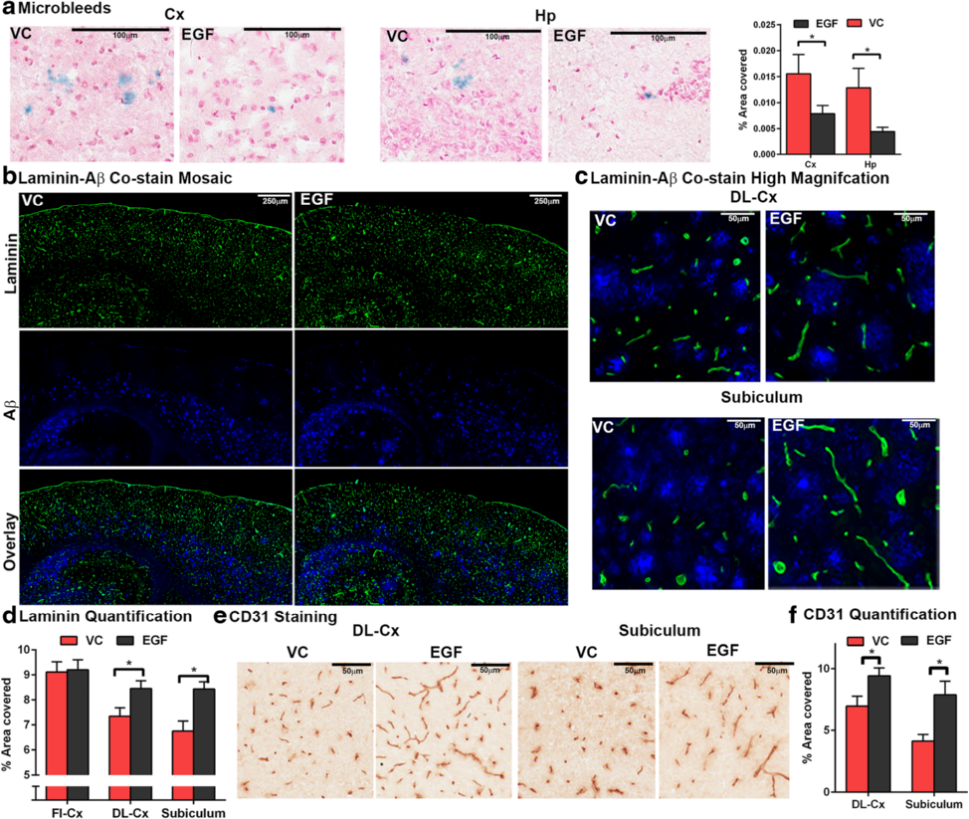
EGF prevents cerebrovascular dysfunction in E4FADF mice. **a** Microbleeds are lower in EGF treated mice (IHC analysis). **b**–**d** Laminin staining is higher in the deep layer cortex and subiculum in EGF treated mice (IHC analysis). **e**, **f** CD31 staining is higher in the deep layer cortex and subiculum after EGF treatment (IHC analysis). *n* = 8 per group. Data expressed as mean +/− S.E.M.*p < 0.05 by Students *t* test

Next the effect of EGF on microbleed coverage was assessed. Microbleeds were lower in EGF treated mice compared to the VC. The percent area covered by microbleeds was 55 % lower in the cortex and 66 % lower in the hippocampus in the EGF treated group (Fig. 4a, Student’s *t*-test). A sub-goal of this study was to determine whether EGF modulates CV coverage in vivo. Thus laminin-Aβ co-staining was conducted (Fig. 4b, Student’s *t*-test). Visually EGF treated mice had a higher area covered by laminin staining than the VC in the deep layers of the cortex and the subiculum (Fig. 4b–c). Quantification of the laminin stain validates this finding as the percent area covered was 15 % (deep layer cx) and 25 % (subiculum) higher than the VC (Fig. 4d). To further dissect the role of EGF on CV coverage, quantitative IHC was conducted for CD31, a brain endothelial cell marker. In EGF treated mice CD31 coverage was 36 % higher in the deep layer cortex and 90 % higher in the subiculum compared to the VC (Fig. 4f, Student’s *t*-test). IHC staining for claudin 5 positive vessels revealed a similar staining pattern as for CD31 (Additional file 1: Figure S1a). Vessel coverage (% area covered) is a combination of density and length. There were no changes in laminin (Additional file 1: Figure S1b) or CD31 density (Additional file 1: Figure S1c) between VC and EGF treated mice. These data support that EGF prevents the lowering of CV length in E4FADF mice. It is important to note that distinguishing CV length and density with the IHC techniques utilized here is not straightforward. The optimal technique for assessing length and density entails three dimensional reconstruction of larger brain volumes with read read-outs for multiple parameters including branching points, major nodes, vessel length between nodes and the distribution of vessel lengths. Therefore, we prefer the term CV coverage for the purpose of this manuscript.

### EGF has no effect on Aβ levels

**Fig. 5:**
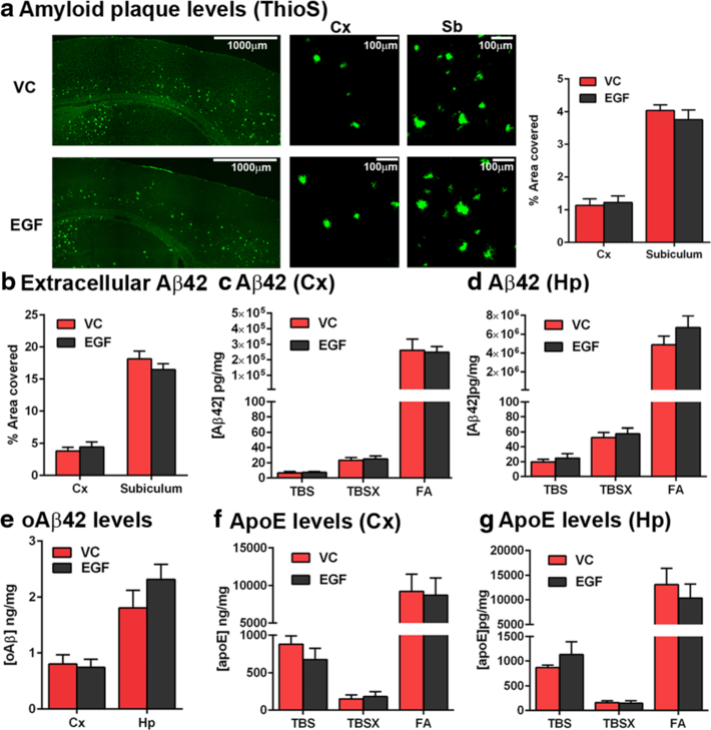
EGF has no effect on Aβ levels. **a**, **b** ThioS plaque and extracellular Aβ levels were the same in EGF and VC treated E4FADF mice (IHC analysis). **c**, **d** When assessed biochemically, EGF also had no effect on soluble, detergent soluble or insoluble Aβ42 levels in the cortex of hippocampus (ELISA). **e** Soluble oAβ levels were unaffected by EGF treatment (ELISA). **f**, **g** ApoE levels were unaffected by EGF treatment (ELISA). *n* = 8 per group. Data expressed as mean +/− S.E.M. Data non-significant by Students *t* test

As EFAD mice are a model of *APOE* modulated Aβ levels and induced dysfunction, we next assessed whether EGF modulates Aβ levels. There were no differences in extracellular amyloid (ThioS, Fig. 5a, Student’s *t*-test) or extracellular Aβ (Fig. 4b–e and Fig. 5b, Student’s *t*-test) staining between the EGF and VC treated mice. EGF also had no effect on soluble, detergent soluble or insoluble Aβ42 levels in the cortex or hippocampus when assessed biochemically (Fig. 5c and d). More recent data suggest that soluble oligomeric Aβ is more AD-relevant than extracellular Aβ, however EGF treatment did not modulate soluble oligomeric Aβ levels (Fig. 5e, Student’s *t*-test). Further, there were no differences in apoE levels between EGF and VC treated mice in any extracted fractions (Fig. 5f–g). Therefore, the protection of EGF on cognitive and CV length was unlikely to be mediated by lowering Aβ42 or changing apoE levels.

## Discussion

AGFs play a key role in controlling total vessel coverage, especially during disease pathology. In AD research the effects that AFGs will induce on AD-like pathology in vivo is a controversial topic. Previously we have demonstrated that EGF can prevent oligomeric Aβ-induced disruption of total vessel length in vitro. Here our goal was to determine whether EGF prevents cognitive and CV dysfunction in an AD-Tg model that incorporates human *APOE* and high Aβ levels, both of which are important for AD and CV function. We demonstrated that E4FADF mice are cognitively impaired, have higher CV leakiness and lower vessel coverage than other groups at 8 months and also had low plasma EGF levels. Critically, we report for the first time that cognitive performance and vessel coverage was higher in E4FADF mice treated with EGF in a prevention paradigm. These data support further research on the development of AGF-based therapies for AD.

### Sex and *APOE4*-induced cognitive and CV dysfunction in EFAD mice

Our data support that sex and *APOE4* impact cognition and CV dysfunction in EFAD mice. In E4FADF the CV dysfunction was accompanied by cognitive dysfunction. In E3FADF mice CV leakiness was higher, CV coverage lower and plasma EGF levels lower compared to EFADM mice. As there were no changes in cognitive function in E3FADF mice compared to EFADM mice, the CV effects may represent early changes that later manifest as cognitive decline. These findings raise important questions on the relationship to human and other in vivo data and the mechanisms underlying sex-induced *APOE* modulated changes in cognition and CV coverage.

In AD patients *APOE4*-induced risk is greater in females [2, 19, 20, 41, 45] consistent with our finding that E4FADF are cognitively impaired compared to all other groups tested at 8 months. In partial contrast a few reports have demonstrated impaired cognition in male *APOE4*-TR mice (which do not overproduce human Aβ) compared to *APOE3*-TR mice [46, 56]. However, in a separate detailed analysis of *APOE*-TR mice the deficits in memory were primarily observed in female *APOE4*-TR mice [21]. Data on CV coverage is lacking in human patients stratified for sex and *APOE* genotype. In 9 and 12 months old *APOE*-TR mice lower microvessel coverage has been demonstrated [1, 4], although the effect of sex is unclear. A detailed comparison of EFAD and *APOE*-TR mice may reveal whether Aβ accelerates the *APOE4* and female sex driven cognitive deficits and changes in CV coverage. In AD microbleeds are associated with male sex, higher blood pressure, lower CSF Aβ42 and *APOE4* [5]. These data contrast with the female bias observed in EFAD mice [12, 20]. A confounding factor is whether additional AD-risk factors are present in male AD patients assessed for microbleeds. For example, level of exercise, hypertension, hyperglycemia, hyperinsulinemia and insulin resistance observed in type 2 diabetes all modulate AD-risk and the CV. Further, men are at a higher risk of developing cardiovascular events. The controlled laboratory conditions utilized here may be more relevant for studying the interaction among sex, *APOE* and Aβ in the absence of additional risk factors. Further research could focus on introducing additional AD-risk factors in vivo.

Females are at a higher risk for AD, which is often attributed to the loss of sex hormones during the menopause [45]. In the mouse model utilized here, with no ovariectomy, there may be inherent sex-differences that modulate CV and AD-like pathology. Extracellular Aβ levels from highest to lowest were: E4FADF > E4FADM = E3FADF > E3FADM consistent with previous reports [12, 51, 61]. Thus, higher levels of Aβ in E4FADF mice could induce or accelerate signaling cascades that cause CV degeneration. As described above CV coverage is lower in *APOE4*-TR mice which supports an acceleration rather than primary causation of CV deficits caused by Aβ. The low CV coverage and EGF levels in E3FADF mice compared to E4FADM mice are not associated with higher Aβ levels, supporting an Aβ-level independent mechanism. Females prior to menopause are at an increased risk for some autoimmune diseases and there are a number of proposed mechanisms underlying these effects [28]. For example, females may have a greater immune/inflammatory response in the periphery and the brain [28]. Although potentially beneficial for fighting infection an enhanced or prolonged immune/inflammatory response in females may predispose, or potentiate Aβ-induced direct and indirect damage to the CV. It is also noteworthy that lower vessel coverage was associated with low plasma EGF levels in EFADF mice. Low EGF plasma appear to increase the conversion of MCI to AD [33] but a sex bias has not been reported. Early reports in mice have demonstrated lower plasma EGF levels in females compared to males [42]. Further, plasma EGF levels in female mice are altered by the circadian rhythm, pregnancy, [53] and are increased with testosterone treatment [52]. Although 17-β estradiol is protective against brain endothelial damage in vitro [38], perhaps the male sex hormones exert a greater protective effect. A possibility is that low EGF levels in females result in lower protection of the CV to damage in AD, which is potentiated by *APOE4* and accelerated by Aβ. Speculating further, the low plasma levels of EGF in female mice may be indicative general growth factor suppression or a form of accelerated aging/senescence.

The effects of *APOE* in AD are multifactorial, complex at the structural and mechanistic level and an area of intense research (reviewed extensively in [23, 34, 37, 43, 55, 59]). On a simplified level apoE4 can effect CV length through modulating Aβ levels and via signaling to cells at the blood-brain barrier (BBB) to alter Aβ-dependent and independent effects. As discussed, there are higher Aβ levels in *APOE4* AD patients and E4FADF mice in this study. Aβ can cause vessel disruption by directly signaling to brain endothelial cells and indirectly via neuronal dysfunction and activation of astrocytes, microglia and pericytes. ApoE also signals to different cells types in the brain to disrupt vessel length. In astrocytes and microglia apoE4 is associated with a detrimental stress-induced (including Aβ) neuroinflammatory response [49]; mediators described as inflammatory can affect brain endothelial function (e.g. cytokines, matrix metalloproteases). In pericytes apoE4 is less effective at suppressing motility [15] and preventing MMP9 production [4, 22], both of which disrupt total vessel length. ApoE4 also directly disrupts brain endothelial cell tight junctions and effects peripheral lipid metabolism to cause brain endothelial dysfunction.

Overall, the female sex-induced, *APOE4* modulated, Aβ accelerated changes in homeostatic signaling at the CV may ultimately converge to predispose and/or amplify stress-induced brain endothelial damage. Our ongoing studies are focused on delineating the role of *APOE*, aging, sex and peripheral risk factors on CV length.

### EGF prevents cognitive and CV dysfunction in E4FADF mice

An important finding here is that EGF prevented CV dysfunction in E4FADF mice. These data are in agreement with the lower EGF plasma levels observed in humans and the beneficial effects of EGF in models of stroke and traumatic brain injury. In contrast improved cognition after blocking the tyrosine kinase activity of the EGF receptor (EGFR) with gefitinib has been reported in drosophilia and AD-Tg mice [57]. Aside methodological differences that commonly impact data (e.g. mouse model, time of treatment), the specificity of gefitinib for the EGFR has been drawn into question. Recently, gefitinib was demonstrated to antagonize a number of G-protein coupled receptors including adrenoreceptors, chemokine receptors, histamine receptors and other neuronal receptors [60]. Gefitinib is reported as a brain penetrant and therefore its beneficial effects in AD-Tg mice could be mediated by non-EGFR targets that play a role in AD-like pathology.

As EGF induced a pronounced effect on preventing cognitive decline in E4FADF mice, it is important to consider potential mechanisms of action. Our data support that the primary mode of action is not on cells within the brain, since brain EGF levels were not altered after EGF treatment. One caveat is that the technique used to measured EGF (ELISA of whole cortex or hippocampus) may have failed to detect regional high concentrations of EGF. Further, EGF did not modulate Aβ levels unlike VEGF in AD-Tg mice [58]. Although VEGF lowers Aβ levels in slice cultures [11] a role for EGF in this process has not been assessed. As EGF did not change Aβ levels, one hypothesis is that EGF acts directly on brain endothelial cells to prevent disrupted signaling induced by the interactive effects of female sex, *APOE4* and Aβ in E4FADF mice. This is consistent with our in vitro data [31], the beneficial effect of EGF in wound healing, the promotion of angiogenesis in cancers that produce high EGF levels or that contain a mutation in erbb2 (a dimerization receptor partner of the EGFR), the protective effect of EGF-induced angiogenesis in stroke models and the general protective function of EGF in epithelial tissue (reviewed in [16]). Further, during the course of treatment EGF may have prevented a loss of key proteins involved in the homeostatic functions of brain endothelial cells, prior to vessel degeneration. For example key proteins involved in the nutrient transport/signaling molecule supply, waste product removal and efflux/metabolic/structural barrier functions of brain endothelial cells. For microbleeds a key question remains on whether EGF prevented global leakiness deficits in capillaries (e.g. via preventing tight junction changes or basement membrane degradation) and/or dysfunction at the post capillary venule level (the site of cellular trafficking).

An alternative hypothesis is that EGF activated receptors outside of brain endothelial cells in the CNS and/or the periphery to cause functional effects that prevent changes in CV coverage. For example, subtle increases in brain EGF levels may induce a beneficial response in pericytes, astrocytes, microglia or neurons to modulate levels of soluble molecules that maintain CV length. In the periphery EGF can signal in cells of virtually every organ in the body [15] and is associated with changes in metabolism and insulin signaling, both of which may protect the CV. An indicator of the peripheral effects is the lower body weight in EGF treated mice. EGF may also prevent cognitive decline independent of any CV changes through the same CNS and peripheral processes described above, in addition to promoting neurogenesis. Our data provide the basis for further studies dissecting if brain endothelial cells are the primary target of EGF.

### CV length and AGFs as potential therapeutic targets in AD

What do the data mean in the context of CV length in AD and therapeutic options for AD patients? There is no clear in vitro or in vivo consensus on whether there is higher or lower CV in AD. One proposal is that lower CV density contributes to AD progression by disrupting cerebral blood flow and the complex transport and metabolic systems of the CV [18, 25, 29, 39]. The counter argument is that AD-pathways induce angiogenesis, causing hypersprouting and tight junction disruption, which increase CV permeability [7, 9, 13]. Traditional angiogenic signaling, typically observed over the short-term is likely not applicable to a chronic neurodegenerative condition such as AD. Pathological angiogenesis, described as the angiogenesis hypothesis [3] may be more applicable for AD: AD-processes not only cause direct vessel degeneration but also induce signaling cascades consistent with increased angiogenesis, however there is a failure to complete maturation and vessel formation. AD-risk factors both in the CNS and periphery could predispose and amplify the disrupted angiogenic signaling in brain endothelial cells.

There is a drive to develop novel strategies for AD as an alternative or complimentary approach for Aβ or tau-based therapeutics. AGF-like drugs may meet this criteria, yet are frequently overlooked in drug development programs. Indeed, an alternative focus is proposed of using therapeutics to block angiogenesis-like processes [54]. Our data with EGF and published data with other factors that increase total vessel coverage support an alternative approach [3, 26, 27, 32], which is to stabilize brain endothelial cells and maintain or increase vessel length. Further research is required to fully dissect the use of AGF-based treatments for AD. Key research areas include the potential for AGFs to reverse AD-like pathology in vivo, distinguishing CV versus peripheral effects and dissecting whether EGF-like molecules act on cells within the brain (i.e., would a brain penetrant AGF-like drug or treatment protocol be more efficacious than non-CNS penetrant). Further research is needed to address the concerns that agonists for AGF receptors could increase cancer risk. Cancer cells themselves are often the origin of high AGF levels; therefore finding a dosing strategy that negates this concern could be important in the elderly AD population.

## Conclusions

In summary, our data demonstrate that EGF prevents cognitive decline in female mice that express *APOE4* and overproduce Aβ. Further, there are lower microbleeds and increased CV coverage but no changes in Aβ levels in EGF treated mice. Developing therapeutic strategies based on AGFs could provide alternative therapeutic options for AD patients.

## Additional file

Additional file 1: Figure S1. EGF had no effect on a laminin or b CD31 density (IHC analysis) in E4FADF mice. Data expressed as mean +/− S.E.M. Data non-significant by Students *t* test. *n* = 8 per group. c. Claudin 5 stained vessels (red) are higher in E4FADF mice after EGF treatment. d Representative full western blot of EGF receptor levels after EGF or VC treatment (the top band was quantified). (PDF 2906 kb)

## Abbreviations

AD: Alzheimer’s disease
AD-Tg: Alzheimer’s disease transgenic mice overproducing Aβ
AGFs: Angiogenic growth factors
ApoE: Apolipoprotein E
Aβ: Amyloid beta
BBB: Blood brain barrier
CD31: Cluster of differentiation 31
CV: Cerebrovascular
EFAD: Mice that express 5xFAD mutations and human APOE
EGF: Epidermal growth factor
EGFR: Epidermal growth factor receptor
EP: End-point
FAD: Familial Alzheimer’s disease
LD: Light-dark box
MCI: Mild cognitive impairment
MP: Mid-point
MWM: Morris water maze
MWM-AP: Morris water maze-acquisition phase
MWM-PT: Morris water maze-probe trial
MWM-VC: Morris water maze-visual cue
NAE: Novel arm entry
NaFl: Sodium fluorescein
NOR: Novel object recognition
OF: Open field
PSD-95: Post-synaptic density protein 95
SA: Spontaneous alternation
VC: Vehicle control
VEGF: Vascular endothelial growth factor
