# Supplementary material for: Epidermal growth factor prevents APOE4 and amyloid-beta-induced cognitive and cerebrovascular deficits in female mice
Source: Acta Neuropathol Commun. 2016 Oct 27;4:111. doi: 10.1186/s40478-016-0387-3 (PMC5084423; doi:10.1186/s40478-016-0387-3)
Supplement: Additional file 1: Figure S1. — EGF had no effect on a laminin or b CD31 density (IHC analysis) in E4FADF mice. Data expressed as mean +/− S.E.M. Data non-significant by Students t test. n = 8 per group. c. Claudin 5 stained vessels (red) are higher in E4FADF mice after EGF treatment. d Representative full western blot of EGF receptor levels after EGF or VC treatment (the top band was quantified). (PDF 2906 kb) [file 40478_2016_387_MOESM1_ESM.pdf]

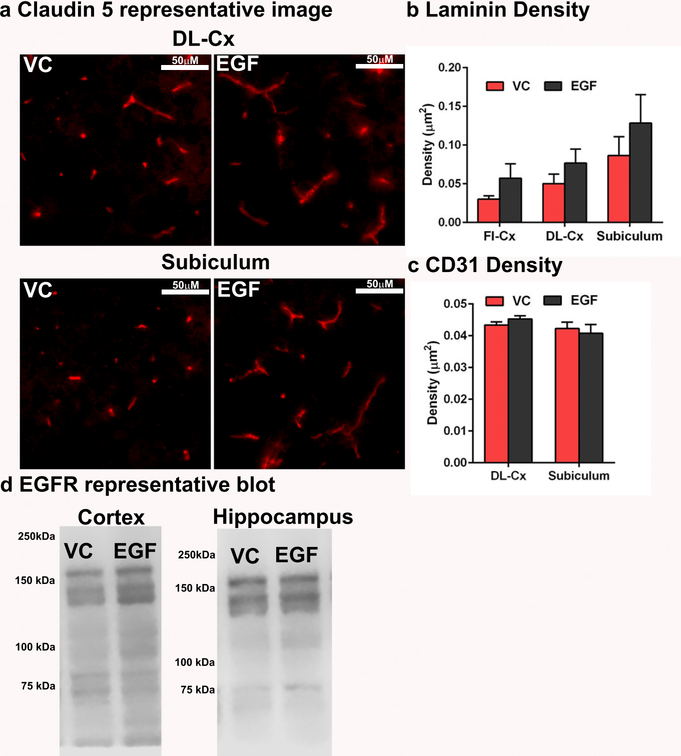
a Claudin 5 representative image
DL-Cx
VC
50μM
EGF
50μM
Subiculum
VC
50μM
EGF
50μM
b Laminin Density
Density (μm²)
0.20
0.15
0.10
0.05
0.00
VC
EGF
Fl-Cx
DL-Cx
Subiculum
c CD31 Density
Density (μm²)
0.05
0.04
0.03
0.02
0.01
0.00
VC
EGF
DL-Cx
Subiculum
d EGFR representative blot
Cortex
250kDa
VC
EGF
150 kDa
100 kDa
75 kDa
Hippocampus
250kDa
VC
EGF
150 kDa
100 kDa
75 kDa
